# Regulation, migration and expectation: internationally qualified health practitioners in Australia—a qualitative study

**DOI:** 10.1186/s12960-020-00514-7

**Published:** 2020-10-07

**Authors:** Melissa Cooper, Philippa Rasmussen, Judy Magarey

**Affiliations:** Adelaide Nursing School, Faculty of Health and Medical Sciences, The University of Adelaide, Adelaide, SA 5005 Australia

**Keywords:** Health practitioners, International, Qualified, Regulation, Migration, Registration, Experiences, Qualitative research

## Abstract

**Background:**

The global movement of internationally qualified health practitioners (IQHPs), seeking to live and work outside of their place of origin, is subject to considerable study and scrutiny. Extensive published material exists, from government enquiries and print news media articles to peer-reviewed papers, reporting on the views and impacts of migration and practitioner registration. Unsurprisingly much of the research focuses on the two largest groups of health professionals, international medical graduates (IMG) and internationally qualified nurses (IQN). This paper presents a unique case study examining the challenges and complexities of navigating the regulatory processes for skilled migration and practitioner registration in Australia.

**Discussion:**

The study comprised a review and analysis of the current policy frameworks, standards and assessment models applied by regulators affecting skilled migration and registration of IQHPs. To target the triangulated themes of regulation, experience and expectations, a phenomenological component was also conducted with the mapping of shared experiences of four key participant groups comprising the following: assessors operationalising the current policies and processes governing skilled migration and registration, educators offering preparatory and training programs to IQHP, workforce agencies engaging with and recruiting IQHP and internationally qualified doctors, nurses and midwives. The study was informed by rich qualitative data extracted from twenty-eight in-depth semi-structured participant interviews. Key themes and points of intersection between participant experiences and the regulatory frameworks were identified using theory and data-driven coding and thematic analysis via the NVivo 12 plus software.

**Conclusion:**

From studying the complexities of the current regulatory processes for skilled migration and practitioner registration and informed by participants with first-hand knowledge and experience, this research found a clear argument for a re-examination and update of the current regulatory requirements for IQHP. Without greater innovation, harmonisation, evidence-based solutions and reform, it is likely that Australian regulators, policymakers, employers, and the nursing, midwifery and medical professions at large will continue to experience challenges in registering, employing and supporting IQHP, while maintaining the safety of the public requiring care in the Australian healthcare system.

## Introduction

Recent trends, reported by the Organisation for Economic Cooperation and Development (OECD) [1] indicate a continued rise of health practitioner migration worldwide. Of the 111 116 medical practitioners registered in Australia in 2016 [2], the OECD assigns 28 283 to international medical graduates (IMG) up from 24 892 in 2012 and 14 808 in 2007 [1]. Of the 386 289 nurses registered in Australia in 2016 [2], the OECD assigns 51 180, a notably smaller increase from 45 364 in 2012 and 38 108 in 2007 [1]. A preliminary review of these numbers would suggest a steady reduction in Australia’s reliance on the recruitment of IMG and IQN to address the 2009 National Health and Hospital Reform Commission projected workforce shortages [3]. This reduction would align with the 2010 call by the World Health Organisation [4], for a more ethical recruitment of health practitioners to avoid sourcing skilled health workers from countries with acute shortages. However, on closer inspection, in terms of absolute numbers, of the OECD countries, Australia had the third largest upward swings in the percentage of internationally qualified doctors and nurses.

Regulatory, statutory and assessing authorities have key roles in facilitating or restricting IQHP access to registration, migration and employment in their trained profession in the country of destination. Host countries, such as Australia, face challenges in maintaining professional practice standards and ensuring the safety of their health care consumers, whilst also accommodating the ever-changing patterns of workforce need through the use of IQHP skills [5]. In an attempt to achieve these outcomes, by imposing particular regulatory or assessment models, authorities may unwittingly penalise those with equivalent overseas qualifications or experience [6]. Consequently, many internationally trained professionals, especially from the Global South, find it difficult to gain registration or migration in the host country due to having to fulfil various long, costly and complex regulatory requirements [6].

An extensive review of the available published material related to skilled migration and registration was undertaken to inform this paper, ranging from government enquiries [7–9] and recent print news media articles [10] to peer-reviewed papers. The literature clearly indicates that the challenges and complexities of migrating, registering and entering workforce co-exist for health practitioners. One study captured a common finding that migration appears ‘a complex and dynamic process of mobility which starts with the initial aspirations and hopes of the migrant, and is never quite over even when the desired destination has been reached successfully [11]’.

In recent years, there has been an increase in the study of lived experiences of IQHP entering the Australian healthcare setting [12–19]. Unsurprisingly, the research focuses on the globe’s two largest groups of health professionals, IMG and IQN, with limited available literature on the migration, registration and integration of internationally qualified midwives [13]. However, there appears to be limited materials available recently examining and comparing the challenges and complexities of IMG and internationally qualified nurses and midwives (IQNM) navigating the regulatory processes for both skilled migration and practitioner registration in Australia. The lack of contemporary research is significant considering the number of government-funded enquiries and reviews [20] and the long-standing Australian immigration policy encouraging the migration of internationally qualified nurses, midwives and doctors, by listing each profession on the skilled occupations list [17].

Regulators and assessing authorities, such as the Australian Health Practitioners Regulation Agency (Ahpra), report [21] that overseas-trained practitioners are subject to rigorous assessment processes to determine whether they have the knowledge, skills and professional attributes necessary to practise their profession in Australia. This case study was designed to examine the veracity of this statement and comparatively analyse two opposing models of assessment within the National Registration and Accreditation Scheme (NRAS) framework for IMG and IQNM. In addition to an extensive review of the literature, the researchers also examined the policy frameworks, standards and assessment models applied the by regulators against the requirements of the National Law [22] and the principles and legislation [23] governing Australia’s General Skilled Migration programme by assessing authorities. For the purposes of this study, regulators included the Medical Board of Australia (MBA) and Nursing and Midwifery Board of Australia (NMBA), and assessing authorities comprised the Australian Medical Council (AMC) and Australian Nursing and Midwifery Accreditation Council (ANMAC). A further review of the structure and delegated roles and responsibilities of the national boards (and their committees) of each regulator and assessing authority was then undertaken to identify the interconnectedness of pathways and processes used to assess IQHP for skilled migration and registration [21]. Whilst the standards for registration and skilled migration appear clear for IQHP, the principles of access, equity and transparency and the claim by regulators and assessing authorities to possess robust assessment processes are not.

## Methods

The case study methodology [24] comprised an examination of the current views, regulatory governance and recommendations affecting skilled migration and registration of IQHPs. Analysis of the policy frameworks, standards and assessment models applied by regulators against the requirements of the National Law [22] and the principles and legislation [23] governing Australia’s General Skilled Migration programme was completed. To target the triangulated themes of regulation, experience and expectations, a phenomenological component was also conducted through the completion of twenty-eight semi-structured interviews conducted with four participant groups. The four groups, outlined within Table 1, comprised the following: assessors operationalising the current policies and processes governing skilled migration and registration, educators offering preparatory and training programmes to IQHP, workforce agencies engaging with and recruiting IQHP, and internationally qualified doctors, nurses and midwives. Interviews were conducted face-to-face and via tele/videoconference across Australia and internationally, from June 2018 to October 2018.

**Table 1:** Participants and data collection

**All groups/participants**	**Inclusion:** 1. Age—over 18 2. Gender—male and female 3. Ethnicity—native speakers of English 4. Locations—nationwide (metropolitan, community and rural and remote)	**Primary interview aims:** Participants were asked to describe personal experiences related to the following: 1. Assessment processes for skilled migration and registration 2. An understanding of the registration and skilled migration requirements/processes for IQHP 3. Points of difference between assessment processes conducted by the regulators and assessing authorities 4. Contexts or situations (positive or negative) which influenced their experiences 5. Opportunities for improvement/harmonisation and the assessors own re-designed assessment processes
	**Exclusion:** 1. Contrary to inclusion	
	**Total participants,** ***n*** **= 28**	
**Group 1: Assessors for skilled migration and registration**	**Inclusion:** 1. Experience—permanent and temporary assessors, employed in the role for no less than 12 months 2. Profession—assessors for skilled migration and registration	**Areas of exploration via contextualised questions:** Assessors determining the suitability of IQHPs for skilled migration and registration were also asked to describe the following: 1. Current responsibilities related to the assessment of IQHP, commencement dates and preparation of their roles and responsibilities aligned to their qualifications and experience 2. Organisational quality improvement strategies, including the following: ­ How their organisations identify and rectify issues related to assessment processes ­ When changes to the processes had occurred ­ And their effectiveness ­ How change is received and implemented ­ Responsiveness to a changing regulatory landscape 3. Opportunities for stakeholder feedback on the assessment processes
	**Exclusion:** Australian Health Practitioner Regulation Agency state-based offices in Tasmania, Australian Capital Territory and Queensland	
	**Group 1 participants,** ***n*** **= 4**	
**Group 2: Internationally qualified health practitioners**	**Inclusion:** 1. Profession—nine nurses, one midwife and five doctors 2. Nationality—including but not limited to the following: United Kingdom, India, China and the Philippines. These have been identified as the top four source countries for IQHP seeking migration and registration by the Australian Nursing and Midwifery Accreditation Council and Australian Medical Council 3. Residential state—onshore and nationwide 4. To reduce the risk of bias, such as survivorship bias, a combination of successful and unsuccessful applications made for assessment for the following: a. Skilled migration with the relevant authority, i.e. Australian Nursing and Midwifery Accreditation Council or Australian Medical Council b. Registration with the Australian Health Practitioner Regulation Agency c. Skilled migration and professional registration in 2011, 2016, 2017 and 2018 only	**Areas of exploration via contextualised questions:** IQHPs navigating through the application processes for skilled migration and registration in Australia in 2011, 2016, 2017 and 2018 were also asked to describe the following: 1 Motivations to move to another country such as Australia 2 Commencement and completion of the processes, e.g. offshore or onshore 3 Sources and types of assistance and support received 4 Consistency of assessment approaches used by the regulators and assessing authorities 5 Transparency, timeframes and associated costs 6 Successful/unsuccessful completion 7 Entering workforce
	**Exclusion:** 1. IQHP residing offshore	
	**Group 2 participants,** ***n*** **= 15**	
**Group 3: Educators of IQHP**	**Inclusion:** 1. Experience—core and temporary individuals, employed in the role for no less than 12 months 2. Profession—educators responsible for upskilling IQHP	**Areas of exploration via contextualised questions:** Educators engaging with and upskilling IQHP were also asked to describe the suitability of IQHP seeking preparatory programmes or referred to bridging programmes.
	**Group 3 participants,** ***n*** **= 5**	
**Group 4: Workforce**	**Inclusion:** 1. Experience—core and temporary individuals, employed in the role for no less than 12 months 2. Profession—health care recruitment and workforce representatives responsible for determining the suitability of IQHP	**Areas of exploration via contextualised questions:** Australian healthcare workforce representatives/agencies engaging with and employing IQHP were also asked to describe the suitability of IQHP for employment and entry into the Australian health care workforce.
	**Group 4 participants,** ***n*** **= 4**	

In accordance with the study’s ethics approval, each participant was approached via an invitation by a third-party organisation. To assist in participant deliberations, each person was provided with access to the approved information sheet and consent form. Personal information of potential participants was provided from the third-party organisations; only once consent had been obtained. The number of interviews, assigned to each of the four groups, was aligned to the specific qualitative research aims, questions and theoretical framework. Recruitment was continued until data saturation was achieved [25]. The years 2011 and 2016–2018 were the focus of this study as the NRAS was introduced on 1 July 2010 for regulating health practitioners across Australia, including doctors, nurses and midwives. The years 2011 and 2016–2018 were also selected as it allowed for a 1-year implementation and a five to seven operational period for the scheme.

The interview questionnaire included semi-structured contextualised questions and areas of exploration which further assisted in addressing research aims and obtaining required data. The questions were applied to understand participant views and experiences (positive and negative) at each stage within the assessment process, timeframes for completing the processes (often commencing whilst applicants are located offshore), associated costs and types/sources of assistance provided and received. Furthermore, data was collected on whether most applicants successfully completed the processes, then registered, migrated and entered the Australian healthcare workforce. Recommendations for improvement were also sought and most significantly whether each participant, including the assessors, could describe the key differences between an assessment for skilled migration and registration

To reduce the risk of unconscious bias, to assist in effective cross-cultural communication [26] and to ensure an understanding of the concept and phases of cultural adaptation [27], the primary researcher completed cultural competence and awareness programmes [28, 29] pre- and post-interviews where participants were asked to share their personal profession-specific experiences. The collection of insightful and rich data was achieved by, often unexpectedly lengthy, interviews where participant’s and researcher’s shared motivation was the opportunity to inform and improve the assessment processes used by regulators and assessing authorities. An unexpected outcome expressed by the IQHP participants was a therapeutic or cathartic experience in telling their individual story through the interview process [30].

## Conceptual framework

A framework [31] for creating a robust codebook assisted in establishing and analysing the interview data. A sequential process was used to create concepts aligned to the models of assessment then code the demographic data and experiences of each of the twenty-eight participants within the NVivo 12 plus software. At key points within the extensive coding process, such as before each participant group was commenced then midway through the process and again at completion, the quantity and relevancy of the concepts were reviewed to ensure alignment to the research aims. Several concepts were amended, amalgamated/extended and duplicates deleted resulting in a framework with a mixture of theory and data-driven themes and ideas. Throughout the month-long coding process, where every hour of interview time took-up-to 5 h to code, the integrity of the data was further assured by cross-checking and comparing all interview transcriptions with over 38 h of audio, expanding abbreviations to full text and repairing over nine hundred discrete sections of inaudible and unintelligible language text. The conceptual framework created from the coded data allowed for a comparison of the models of assessment, the identification of points of intersection and assisted in analysing the research data to validate theories on the complexities of navigating each process.

**Fig. 1:**
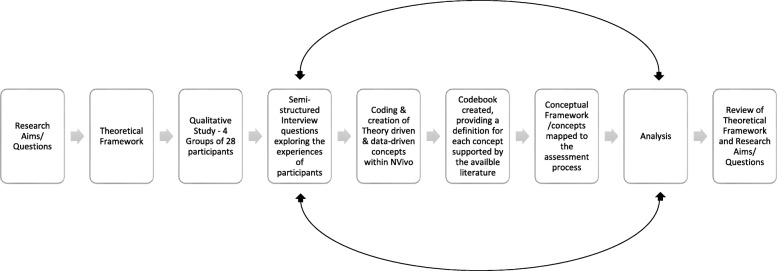
The research pathway

An interpretive phenomenological approach [32] was used with recognition that ‘participants hold the power of knowledge since they are the only experts with the lived experience’ [33]. At specific points within data collection, namely participant interviews, verbatim transcription, and theory and data-driven coding [31], the primary researcher documented non-verbal information, areas for further investigation or data generation, and personal experiences about each unique participant encounter. A reflective journal was also created to assist the primary researcher in debriefing from the often-difficult realities (Fig. 1).

## Results

Using NVivo’s analytical capabilities, from simple word searches to the more complex content matrices and matrix query tables, clear and immediate patterns appeared in the data validating the anticipated research results within the three key themes of regulation, experience and expectation. Multiple points of common intersection were identified, as participants described the processes as complex, duplicated, expensive, inconsistent and challenging. Participants in this case study also highlighted that familiarisation of one process through un/successful navigation then influences the experiences navigating the other. These preliminary findings supported further interrogation of the assessment processes for both skilled migration and registration, with each regulatory practice currently failing to meet the expectations of the participants across all four groups.

Based on the intersecting commonalities, within all twenty-eight participants interviews, six sub-themes emerged from the rich data, comprising expectations, cultural orientation, harmonisation, communication, workforce demand, and education, assessment and accreditation. With the aim to address and improve the multiplicity and complexities of assessment processes and outcomes for IQHP, the data contained within these key concepts will be used to create a set of recommendations which will, for the first time, be critically informed by those who are directly responsible for: operationalising the current policies and processes governing skilled migration and registration, delivering preparatory and training programmes to IQHP, and engaging with and recruiting IQHP and critically the internationally qualified nurses, midwives and doctors.

In addition to the shared dream of moving to Australia and the intersection of shared experiences related to navigating the assessment processes, each of the fifteen IQHP exhibited common personal attributes including resilient, persistent, motivated, resourceful and adaptable. Furthermore, all IQHP described experiencing stages of cultural adaptation [34], declaring feelings of isolation, shame, hope and hopelessness, fear, culture shock, lack of cultural safety, racism, financial hardship, professional displacement and significant impacts on their mental health. One participant describing a deeply personal experience of a fellow IQHP taking their own life as a result of a failure to gain registration and employment as a health practitioner in Australia. These participants’ stories will be presented in a subsequent paper which will provide the audit trail for the findings presented here.

## Discussion

In a 2018 [35] discussion paper by the United Kingdom’s Professional Standards Authority on the perspectives of international regulators applying the six principles of right-touch regulation—proportionate, consistent, targeted, transparent, accountable and agile [36]—Ahpra, along with nine other regulators, stated that they had applied the principles of right-touch regulation to help them overcome the problems and challenges faced by their organisations [36]. Further, Ahpra justified the move to a risk-based approach as one of the solutions to effectively address and manage the number and complexities of practitioner notification, as well a number of planned operational changes and the overall strategic plan which considers the principles of right-touch regulation. However, other than stating that the assessment of IQHP was one of Ahpra’s legislated functions, none of the key problems highlighted in this case study was acknowledged or indeed overcome for multiple assessment pathways operationalised for 16 regulated health profession groups across Australia. The results informing this paper reported an opposing view to Ahpra, with participants describing a range of long-standing complex problems still to overcome.

In 2019, the AMC and ANMAC, two of the largest assessing authorities out of the 18 operating in Australia, reported continued growth in the number of IQHP seeking assessments, e.g. ANMAC completed over 6400 assessments for skilled migration in its 2018/2019 financial year with a total revenue of $1 984 380, while the AMC reported a total revenue of $18 265 384 for the examination fees of IMG in 2018/2019 and 5052 portfolios created [37]. The transparency of revenue earned by these two authorities, a request in the most recent review into the NRAS [9], is critical when evaluating the impacts on IQHP. By comparison, the revenue declared by Ahpra, the MBA and NMBA does not publicly report on the apportioned income derived from overseas assessments for registration.

A review of revenue accrued by regulators and assessing authorities through the application of models of assessment when compared with the experiences and cost born by IQHP is particularly relevant when considering completion rates. The data on how many applicants are unsuccessful in gaining registration/skilled migration and make an undefined number of attempts to successfully complete the process is unidentifiable. The AMC reported that the pass rate for IMG undertaking their clinical exam in 2018/2019 was only 21.7% or a total of 1978, with several IMG re-presenting and re-siting the tests and just under 50% presenting for the first time. Notably, there is a much higher success rate for the workplace-based assessment with 123 successful completions from 125. Furthermore, there appears no publicly available data on the number of applications received by Ahpra from any IQHP across the 16 regulated professions.

The national registration boards and accreditation authorities assert that the two application processes for determining suitability for registration and skilled migration are and should be entirely separate with each organisation possessing a discrete role and function governed by independent legislation [22, 23]. The NMBA and ANMAC caveat their separate functions by advising IQNM that successful completion in one application does not guarantee success in the other [38]. (Table 2).

**Table 2:** Legislative functions for IQHP assessment

MBA and NMBA	MBA/AMC and ANMAC
Authorised under the Health Practitioner Regulation National Law Act (as in force in each Australian State and Territory) to assess an applicant’s eligibility for registration as a doctor, registered nurse, enrolled nurse or midwife in Australia. Standards must be approved by the Council of Australian Government - Ministerial Health Council (for the National Registration and Accreditation Scheme).	Authorised under the Migration Regulations 1994, Migration (LIN 19/051: Specification of Occupations and Assessing Authorities) Instrument 2019, to assess an applicant’s eligibility for the general skilled for migration as a doctor, registered nurse or midwife in Australia. Standards must be aligned to the provisions articulated by the Department of Home Affairs (DHA) [formerly the Department of Immigration and Border Protection] [39].

Whilst the four key participant groups confirmed an entirely separate assessment process, many participants described frustration with the requirement to submit and assess duplicate evidence against the same criteria applied for registration and skilled migration, comprising proof of identity, English proficiency requirements, educational equivalence, recency of professional practice and fitness to practise and indemnity insurance. For the IMG, these requirements may extend further to completing parts 1 and 2 of the AMC process and provide additional evidence for limited registration with supervised practice.

Whilst the regulators and assessing authorities advocate for separate processes, only two assessors within group 1 and one IQHP could provide a key point of difference—professional references. This research found no clearly justifiable point of difference between the standards, criteria and process used by the regulator and assessing authorities when charging and assessing IQHP for registration and skilled migration. However, the authenticity of the information provided within the reference, contributing to an assessment of the IQHP skill, is difficult to ensure.

## Future directions

In 2020, the NMBA/Ahpra is introducing changes to the current Australian registration requirements. These changes which will have significant implications for all internationally qualified registered nurses, midwives and enrolled nurses, as it will replace the current process operationalised and experienced by research participants. A new outcomes-based assessment (OBA) model will set a new framework for how competency to practise is assessed and ensured. The model has been more than 5 years in the making, with the tendered contract project initially due to commence in September 2014 and complete September 2015 [40]. The NMBA defines OBA as: ‘assessing what the nurse or midwife should be capable of doing’. This means measuring the nurse or midwife’s knowledge, skills and attributes against the relevant NMBA standards for practice, previously termed national competency standards [41].

The OBA model aligns to the regulatory frameworks used both nationally, by several other profession-specific groups—particularly medicine—and internationally by countries such as New Zealand, Canada, the United Kingdom, Ireland and South Africa. However, the decision by the NMBA to follow the MBA down a pathway where overseas-qualified practitioners are required to complete an exam (National Council Licensure Exam for IQNM and Multiple Choice Question exam for IMG) is likely to lead to many of the same process issues highlighted in this paper as well as a new risk of the introduction of unaccredited/regulated preparatory courses to replace the already costly bridging programmes offered to upskill practitioners with non-equivalent entry-to-practice qualifications and experience.

Notification of the NMBA’s planned transition to the new OBA model was made publicly available [42] by Ahpra from 2014. However, a common theme highlighted in this paper regarding a lack of regulatory transparency appears to remain with limited/changing information made publicly available regarding proposed assessment charges, transition time frames/defined end dates to approved bridging programmes, key stakeholder and consumer consultation on the model and most significantly research literature and evidence to support the change. Finally, and as identified in the recently published Commonwealth Report [41] of the Review of Nursing Education, ‘Australia will simultaneously use two diametrically opposite approaches to determine suitability for practice—outcome-based individual assessments for nurses educated abroad and input-based institutional accreditation for nurses educated in Australia’.

## Limitations

Overall, the literature retrieved and reviewed to inform this paper could have been wider ranging as it excluded records published before 2008 and did not allow for retrieval of information located: within foreign language literature or additional health-related databases, through personal approaches to experts in the field to find unpublished reports or via regulatory authorities/systems in countries outside those selected. Furthermore, in accordance with the ethics approval for this study, strict limitations were placed on participant inclusion and exclusion criteria (listed in Table 1). Although this requirement assisted in ensuring a focus on the aims and objectives of the research, it limited the study to a defined set of criteria. It should also be noted, despite several formal requests, the researchers were unable to secure participation of assessors (for group 1) from the multiple case workers employed at Ahpra throughout the pre-determined jurisdictional State and Territory offices.

To build on the literature review undertaken as part of this research project and to assist in ensuring contemporary, consistent and accurate information was presented in this paper, a formal data request was made to Ahpra, in December 2018, for a copy of the literature review undertaken to inform the new model of OBA for IQNM. However, the request was declined in July 2019, with the NMBA/Ahpra determining the review was an internal organisational document that could not be provided/published externally as it provides the regulatory foundation and evidence base of the new model of assessment for IQNM. Further, as described in the ‘Future directions’ section, it should be noted that the statistical data published by Ahpra or the profession-specific boards, including the MBA and NMBA, does not include details of the number of completed applications for overseas assessments or successful/unsuccessful applications for professional registrations of internationally qualified doctors, nurses and midwives.

Finally, it should be acknowledged that although this paper provides systematically assembled, quality appraised and appropriately synthesised information to guide changes to the policies and guidelines related to the governance of IQHP, the scale and limitations would suggest value in conducting a larger-scale prospective study examining the area of regulation and IQHP.

## Conclusion

The process of changing government policy is inherently political [43] and so to exert influence requires a sound understanding of the policy and an ability to engage actively with it. Furthermore, complex changes to policy require a whole-of-government approach, as no single organisation/agency/government department has all the pieces of the puzzle [44]. Changing mindsets of organisations and people involved in the operationalisation of assessment models is as important as a change to the policy [44] or regulatory practice. The findings obtained through this research clearly support the argument for a re-examination and update of the current regulatory requirements for IQHP. Greater innovation, harmonisation and evidence-based solutions are required to support and reform the standards, guidelines and policy which are used to regulate IQHP. Without this, it is likely that Australian regulators, policymakers, employers and the nursing, midwifery and medical professions at large will continue to experience challenges [5] in registering, employing and supporting IQHP, whilst maintaining the safety of the public requiring care in the Australian healthcare system.

## Data Availability

The data generated, analysed and supported the findings of the current study are held by the University of Adelaide, but restrictions apply to the availability of this information, which were used under the ethics approval for the current study, and so are not publicly available.

## Abbreviations

Ahpra: Australian Health Practitioners Regulation Agency
AMC: Australian Medical Council
ANMAC: Australian Nursing and Midwifery Accreditation Council
DHA: Department of Home Affairs
IMG: International medical graduates
IQHP: Internationally qualified health practitioners
IQNM: Internationally qualified nurses and midwives
MBA: Medical Board of Australia
NMBA: Nursing and Midwifery Board of Australia
NRAS: National Registration and Accreditation Scheme
OBA: Outcomes-based assessment
OECD: Organisation for Economic Cooperation and Development
